# Multiple sclerosis and the microbiota: Progress in understanding the contribution of the gut microbiome to disease

**DOI:** 10.1093/emph/eoac009

**Published:** 2022-06-13

**Authors:** Hendrik J Engelenburg, Paul J Lucassen, Joshua T Sarafian, William Parker, Jon D Laman

**Affiliations:** Department of Pathology and Medical Biology, University Medical Center Groningen, Groningen, The Netherlands; Brain Plasticity Group, Swammerdam Institute for Life Sciences, University of Amsterdam, Amsterdam, The Netherlands; Brain Plasticity Group, Swammerdam Institute for Life Sciences, University of Amsterdam, Amsterdam, The Netherlands; Center for Urban Mental Health, University of Amsterdam, Amsterdam, The Netherlands; Department of Biology, Duke University, Durham, NC, USA; XPara Mutualistic Helminths, LLC, Durham, NC, USA; Department of Pathology and Medical Biology, University Medical Center Groningen, Groningen, The Netherlands

**Keywords:** nutrition, multiple sclerosis, gut microbiome, gut microbiota, diet

## Abstract

Multiple sclerosis (MS), a neurological autoimmune disorder, has recently been linked to neuro-inflammatory influences from the gut. In this review, we address the idea that evolutionary mismatches could affect the pathogenesis of MS via the gut microbiota. The evolution of symbiosis as well as the recent introduction of evolutionary mismatches is considered, and evidence regarding the impact of diet on the MS-associated microbiota is evaluated. Distinctive microbial community compositions associated with the gut microbiota of MS patients are difficult to identify, and substantial study-to-study variation and even larger variations between individual profiles of MS patients are observed. Furthermore, although some dietary changes impact the progression of MS, MS-associated features of microbiota were found to be not necessarily associated with diet per se. In addition, immune function in MS patients potentially drives changes in microbial composition directly, in at least some individuals. Finally, assessment of evolutionary histories of animals with their gut symbionts suggests that the impact of evolutionary mismatch on the microbiota is less concerning than mismatches affecting helminths and protists. These observations suggest that the benefits of an anti-inflammatory diet for patients with MS may not be mediated by the microbiota per se. Furthermore, any alteration of the microbiota found in association with MS may be an effect rather than a cause. This conclusion is consistent with other studies indicating that a loss of complex eukaryotic symbionts, including helminths and protists, is a pivotal evolutionary mismatch that potentiates the increased prevalence of autoimmunity within a population.

## INTRODUCTION

Multiple sclerosis (MS) is a chronic inflammatory disease characterized by inflammatory activity in the central nervous system. Clinical symptoms range from motor dysfunction to cognitive deficits, and its typical neuropathological changes include both de- and re-myelinating lesions, the increased presence of lymphocytes in the brain, widespread inflammatory changes, gliosis and axonal degeneration [1]. The disease is very common, affecting more than 700 000 individuals in the USA alone [2]. With costs of treatment exceeding $60 000 per person per year in the USA [3], the condition has become a public health crisis (Box 1).

**Box 1. Societal impact of multiple sclerosis**

MS is an incapacitating, degenerative condition of the nervous system, characterized by a strong neuro-inflammatory component. Its symptoms and progression vary widely between people and as a result, MS represents an unpredictable life-long condition with major implications for the quality of life of patients and their families.

- Since 2013, the number of people living with MS has increased worldwide from 2.3 million to 2.8 million [83].
- Prevalence in the USA is 2.8 times higher in females compared to males [2].
- There are 30 000 children (younger than 18 years) diagnosed with MS worldwide, although onset of MS is normally during adulthood [83].
- Incidence of MS varies largely between studies and regions. In the USA, annual incidence of MS ranges from 0.15 to 7.5 cases per 100 000 people [84].
- Although an increase in prevalence is likely in part due to longer survival of MS patients, incidence is also rising, particularly in females [85].
- Minimal costs of current disease modifying therapies (DMTs) for MS are $50 000 annually [3].
- Costs of first-generation DMTs rose between 21 and 36% per year [3].
- Through large and increasing profit margins, current MS treatment costs are a heavy and unsustainable economic burden on people with MS in the USA [3].

**Box 2. Nutritional science: hurdles and limitations**

Nutritional science can be of great benefit to public health, but is hampered by some large hurdles and limitations, as reviewed by Weaver and Miller in 2017 [86]:

**Dietary composition**

- Important is not only which nutrients are supplemented, but also which are displaced.
- Appeal of dietary composition in a study will affect adherence and dropout rates.
- Effectiveness of dietary intervention can be dependent on the baseline dietary pattern of participants, and variation therein.

**Randomized controlled trials**

- The reductionist approach of randomized controlled trials in nutritional research is questionable, as food patterns shift over time and location [87].
- Comparing an interventional diet to a diet that is not deprived of the nutrients that are studied might not accurately study the effect of the nutritional intervention of interest.
- Depriving the control group of a nutrient is unethical and might induce harm. When recruiting participants on the basis of nutritional deficiencies due to their own preferred diet, it is ethically questionable to leave these participants untreated.
- Blinding of research is challenging in nutritional research. Participants know what they are eating. Still, blinding for researchers that collect and examine primary outcomes should be possible.

**Time**

- Concerning MS, which is a chronic and lifelong disease, a longitudinal study from onset to elderly age would be preferred as this would most accurately unravel disease outcomes. However, this is practically unattainable because of low adherence, high dropout rates and labor intensity and often a lack of funding.

Among many hypotheses, MS has been proposed by some to be a consequence of ‘evolutionary mismatch’. As with other autoimmune conditions, the induction and progression of MS has been attributed to the absence of complex eukaryotic symbionts, such as helminths and protists [4–10]. The loss of these previously ubiquitous symbionts is a consequence of ‘systems hygiene’ factors, such as sewer systems, water treatment facilities and food processing plants, that were employed after the industrial revolution as an effective means of preventing infectious disease [11]. Other evolutionary ‘mismatches’ proposed to be implicated in the high rates of MS in Western society, include vitamin D deficiency [12] due to indoor work environments, heavy smoking [13] due to the ready availability of cultivated and processed addictive plants, and chronic psychological stress [14] caused by a wide range of issues inherent in Western culture [15]. Finally, the Western diet, high in saturated fats and processed carbohydrates, is considered a key evolutionary mismatch for more than 70 years and a possible contributing factor for MS [16, 17]. However, studies on the impact of nutrition on disease are challenging (Box 2), and many questions remain to be addressed.

Although evolutionary mismatches could be seen as a prerequisite for the initiation and possible development of MS, host immune function likely plays the most substantial role in determining which individuals acquire MS and which do not. Based on genetic risk factors for MS, specific cell subsets of both the acquired and innate immune system contribute to the onset and progression of MS [18]. In addition, it seems likely that viral infection(s) and presumably the accompanying immune stimulation can serve as a trigger for the onset of MS [13]. Thus, evolutionary mismatches could lay the necessary groundwork for MS, whereas a complex array of factors associated with the subsequent and/or aberrant host immune function could ultimately play a major role in the pathogenesis and progression of the disease.

The human body is composed of a myriad of symbiotic organisms in addition to human-derived cells. This has led investigators to look at other factors that may be involved in MS pathogenesis. Emerging evidence suggests that, in particular, the gut microbiota, which involves the combined communities of microorganisms, like bacteria, archaea, protists, fungi and viruses in the gut, could play a role in the pathogenesis and progression of MS. The microbiota and the relative proportions of commensal, symbiotic and pathogenic microorganisms are likely to be affected by evolutionary mismatches. In particular, the pro-inflammatory Western diet has long been thought to be a factor in the onset and progression of MS, as MS prevalence was observed to be correlated with per capita consumption of fats and oils, protein, and calories [16, 17]. Furthermore, the gut microbiota is strongly affected in experimental autoimmune encephalomyelitis (EAE), a laboratory animal model for aspects of MS [19–21]. For example, fecal microbiota transplantation from healthy mice to mice with EAE resulted in less severe pathology compared to mice that did not receive a fecal microbiota transplant [19]. These fecal transplant-induced improvements in disease progression appeared to be mediated by decreased immune system activation in the brain as well as by increased protection of the nervous system from damage [19]. In addition, patients with MS also tend to have differences in their microbiota composition compared to control subjects [22]. These observations, taken together, provide reason for optimism regarding the possibility that modulation of the gut microbiota may offer a possible treatment approach for MS.

We will discuss emerging evidence regarding the role of the microbiota in the pathogenesis and progression of MS. Particular attention will be paid to dietary interventions, changes to the microbiota and the effects on the progression of MS. Focus will also be directed toward the evolution of symbiosis within the gut and what is known regarding the impact of evolutionary mismatches on that symbiosis.

## AIMS AND APPROACH

In this narrative review, we evaluate nutritional interventions in MS and how these studies inform us about the role of the microbiota in MS. In addition, the effects of standard pharmaceutical-based interventions on the microbiota were considered with respect to the progression of MS. Finally, we evaluated what is known regarding the evolution of symbiosis in the gut, considering the emergence of evolutionary mismatches that affect those symbiotic relationships. We incorporate general literature, relevant research-articles, clinical trials and (systematic) reviews that were found manually through Google Scholar, PubMed, Web of Science, clinicaltrials.gov and EudraCT. Emphasis was placed on the composition of the gut-microbiome in MS patients, and information regarding immune-related functions of relevant microbiota was considered. Key terms of this article are defined in the Running Glossary shown in Box 3.

**Box 3. Key definitions—Running Glossary**

**Adaptive immunity** is the ‘Second line of defense by lymphocytes using somatically rearranged receptors that can recognize any antigen and are initially slow (within days) before immunological memory is formed.’ [33]

**Complex eukaryotic symbionts** are typically macroscopic and include helminths and protists that form symbiotic relationships with humans living in regions without widespread systems hygiene [11]. Fungi, although eukaryotic, are microscopic, are not depleted by systems hygiene and are included with the microbiota rather than in this category.

**Dysbiosis** is an imbalance in microbiome composition [88] or a change in normal microbiota [89]. Here, dysbiosis is defined as a change from the normal microbiota, which can result in pathogenic effects. It must be stated that the concept of healthy human microbiota is not yet clearly elucidated [90]. In this case, dysbiosis refers to taxonomic and functional changes in the gut microbiome of MS patients compared to controls.

**Experimental autoimmune encephalomyelitis** is ‘a model of the neuroimmune system responding to priming with central nervous system (CNS)-restricted antigens. It is an excellent model of post-vaccinal encephalitis and a useful model of many aspects of multiple sclerosis’ [91].

**Evolutionary mismatch** refers to disease-associated differences between modern lifestyles/environments and the conditions under which traits originally evolved [92].

**Holobiont** is ‘a unit of biological organization composed of a host and its microbiota’. The previously used definition [93] should be amended to include complex eukaryotic symbionts such as helminths and protists.

**Hologenome** is ‘the complete genetic content of the host genome, its organelles’ genomes, and its microbiome’ [93].

**Innate immunity** can be defined as immunity that does not rely on adaptive memory but rather is genetically programmed and attempts to detect evolutionary conserved patterns. Its mechanisms include physical barriers, chemical signals and some types of immune cells [33].

**Microbiome** refers to ‘a characteristic microbial community, occupying a reasonable well-defined habitat, and which may have distinct physio-chemical properties and effects on its host. The microbiome not only refers to the microorganisms involved but also encompass their entire theatre of activity, which results in the formation of specific ecological niches. The microbiome, which forms a dynamic and interactive micro-ecosystem prone to change in time and scale, is integrated in macro-ecosystems including eukaryotic hosts, which are crucial for their functioning and health’ [94, 95].

**Microbiota** are microorganisms such as bacteria, fungi and archaea, also viruses, which inhabit and colonize an organism, such as humans [96].

**Postbiotics** are ‘compounds produced by microorganisms, released from food components or microbial constituents, including non-viable cells that, when administered in adequate amounts, promote health and well-being’ [97].

**Prebiotics** are ‘substrates that are selectively utilized by host microorganisms and thought to restore a disturbed balance and may confer a health benefit’ [98].

**Probiotics** are ‘live microorganisms that, when administered in adequate amounts, confer a health benefit on the host’ [99].

**Synbiotics** are ‘a mixture comprising live microorganisms and substrate(s) selectively utilized by host microorganisms that confers a health benefit on the host’ [100].

## OVERVIEW: THE MICROBIOTA, THE GUT BRAIN AXIS, IMMUNE FUNCTION AND MS

Systematic use of evolutionary theory provides a valuable framework for a rational understanding of the gut microbiota and its role in immunity and MS development. For example, evolutionary theory predicts that effects of the host on the microbiota are critical for microbiome composition and function, at least as much as for the well-studied impacts of the microbiota on the host. Although a detailed discussion is beyond the scope of the current review, Box 4 provides a selection of some important considerations. The interested reader is referred to two most useful reviews; Suzuki and Ley (*Science* 2020) particularly focus on genetic adaptation of the human host, discussing evidence for an evolutionary selection in relation to the microbiota [23], while Foster et al. (*Nature* 2017) apply evolutionary and ecological theory to provide a systematic analysis on microbiota in human hosts, plants and even corals [24].

**Box 4. Evolutionary features of (gut) microbiota and human genetic adaptation**

**Ecological and evolutionary aspects**

- Hologenome theory of evolution: reciprocal selection between partners, i.e. host–microbial coevolution, where selection acts on the host and its symbionts as a unit promoting microbial functions enhancing fitness. For instance, formation of organelles such as the mitochondrion in eukaryotes, and endosymbionts of insects.
- Natural selection acting on the gut microenvironment is predicted to favor the evolution of molecular mechanisms that support both host and symbiont. As an example of such a mechanism, the production of a mucus-rich intestinal glycocalyx by the host and expression of mucus receptors by the microbiota creates a nutrient-rich substrate upon which symbiotic microorganisms can effectively grow, and provides the host with close proximity to a microbial community that aids in various host-critical processes such as metabolism and immune development.
- Evolutionary theory predicts that host-to-microbe effects are critical to microbiome composition and function, as least as much as the well-studied impacts of microbe-to-host.
- The (human) host is under ‘strong natural selection to shape the microbiota from the top down and foster a community that is beneficial’.
- ‘Evolutionary theory does not predict that each symbiont strain will provide a benefit, but it does predict that all strains will be effective competitors.’ The outcome of this competition on the gut microenvironment will depend mostly on diet, and to a lesser extent on host genetics.
- Ecological stability of the gut microbiota is a desirable trait in itself, but also a barrier to therapeutic intervention. FMT is a major ecological manipulation to probe the stability of the system.
- Selection of gut microbiota may occur at species level or other taxon levels, but also may occur at the level of physiological functions.
- Selection acting on microbiota-encoded processes can benefit the host without the need for the host to evolve its own adaptive mutations. For instance, gut microbiota from several Asian populations can enzymatically degrade glycans from seaweed, providing the host with nutrition from what would otherwise be indigestible material.

**Insights from non-human primates and Great Apes**

- Evolutionary aspects of MS and the lack of evidence for this disease spontaneously occurring in non-human primates and Great Apes have been discussed in detail by ‘t Hart [143] and Bove [144].
- Human ecology is more important than phylogeny evidenced by convergence of human and 18 species of Old World monkeys’ gut microbiomes [101], emphasizing the critical role of the diet.
- Contrary to expectations based on evolutionary relatedness, the gut microbiome from humans living under non-industrialized conditions resembles that of African Old World monkeys. In addition, these non-industrialized human populations and wild cercopithecine monkeys with eclectic diets have high gut microbiome diversity, higher both than industrialized humans and wild primates with fiber-rich diets [102].

**Dietary adaptations in the human host**

- Host enzymes as well as microbial enzymes can both metabolize some dietary components, such as fatty acids and alcohol.
- Lactase persistence (LP) and non-persistence (LNP, the ancestral condition) may be co-determined by *Bifidobacterium* producing beta-galactosidase.
- Increase in the amylase copy number associated with a shift in low to high starch diet, is likely affected by the genus *Ruminococcus* fermenting resistant starch.
- LC-PUFA can be synthesized by bacteria from at least 10 phyla. For instance, *L. plantarum* can metabolize plant precursors.
- Alcohol resistance: both human enzymes and gut microbiota bacterial enzymes can convert ethanol to acetaldehyde.
- Body mass index (BMI) and metabolic syndrome are associated with the highly heritable taxon of *Christensenellaceae* of the human gut.
- Akkermansia, a mucin degrader associated with BMI, is a heritable taxon, and very recently *A. muciniphila* peptides have been eluted from HLA-II risk alleles for MS [35].

Notes: Many points above are based on Suzuki and Ley [23] and Foster et al. [24], oftentimes by literal or paraphrased quotes, and on references cited therein. Other references are indicated in the Box itself.

**Box 5. Overview of nine nutritional interventions assessed**

**Mediterranean diet** consists of fish, olive oil, vegetables, whole grains and legumes/nuts. This diet is high in fiber and mono- and polyunsaturated fatty acids [50].

**High dietary fiber** means a large amount of fiber in the diet, possibly supplemented. Fiber is plant material that cannot be digested through enzymatic digestion [103].

**SCFA supplement** are supplements of short chain fatty acids (SCFA) such as butyrate [104], propionate [105] and acetate [106].

**LCPUFA supplements** are supplements of LCPUFA or their precursors such as eicosapentaenoic acid (EPA) or docosahexaenoic acid (DHA) [107].

**Paleolithic diet** is based on the food-intake of ancient hunter-gatherers and consists of vegetables, fruits, nuts, seeds, eggs, fish and lean meat, with little consumption of grains, dairy, salt and refined sugar [51].

**Low fat diet**, **s**uch as the Swank diet [57] or the vegan McDougall diet [56] are characterized by their low amounts of saturated fats.

**Ketogenic diet** consists of a high amount of fats and a low amount of carbohydrates, resulting in ketosis [108].

**Biotin supplements** are supplements to increase the serum levels of the vitamin biotin, also known as vitamin B_7_ or vitamin H [61, 62].

**Vitamin D supplements** are supplements to increase serum levels of vitamin D [64, 65].

**Box 6. Recent highlights promoting research on diet and MS**

- Exploration of associations between human genetic variants and the gut microbiome in a large-scale, multi-ethnic cohort [48] shows that host-factors influence the gut microbiome.
- Gut microbiota have a considerable influence on systemic immune cell dynamics [109].
- Establishment of the best approximation thus far of the normal healthy gut microbiome by metagenomic sequencing of over 8000 donors [110]
- Confounders in many microbiota studies can affect taxa-level findings in case-control studies. These include stool quality, BMI, age, red wine consumption and salt intake [111].
- Identification of *Akkermansia muciniphila* peptides presented by HLA-II risk alleles for MS [35].
- Fecal Microbiota Transplant/Transfer (FMT) from strictly organized and controlled feces banks is ‘a major ecological manipulation that may provide valuable insights’ into how to make a microbial community temporarily susceptible to invasion’ [24]. FMT for neurological disease has recently been reviewed in great detail [112].
- Commensal reactive IgA+ plasma cells and plasma blasts are a source of IL-10 in EAE [113].
- Meningeal IgA+ cells that originated from the gut can protect the central nervous system from blood-borne pathogens to enter the brain at venous sinuses [114].
- Very recent studies of twins concordant or discordant for MS allow mechanistic insights into immune mechanisms underlying disease development and contributions of the gut microbiota [115–117].
- Innovation of the non-human primate marmoset (*Callithrix jacchus*) EAE model for microbiota and dietary intervention studies. This includes: (i) First comprehensive typing of the marmoset gut microbiota in health and during the course of EAE [118]; (ii) Dietary intervention with a yogurt-based supplement limiting clinical disease and spinal cord pathology [118]; (iii) Quantitative assessment of the marmoset counterpart of EBV, CalHV3 in distinct anatomical compartments and its modulation by diet and finally, (iv). Development of the gut ecology framework to interpret and apply dietary intervention in the context of immune mechanisms [119].
- Mice deficient in interleukins 17A and 17F had an altered microbiome and did not develop experimental autoimmune encephalomyelitis (EAE). Therein, IL17A/F modulate intestinal homeostasis and facilitate autoimmunity in the CNS. Furthermore, reconstitution of the microbiota to the normal state restored EAE, demonstrating that IL-17 controls central nervous system autoimmunity through the intestinal microbiome [120].
- Gut CD4+ T cell phenotypes are a continuum driven by microbes, and do not consist of strictly demarcated T-helper cell archetypes [121].

The gut microbiota are connected in unique ways to the brain via, among others, the gut–brain axis and the immune system, and these connections play a profound role in both health and disease [25–28]. This paradigm is perhaps not surprising given that the immune system strongly affects the brain, and that in turn, the development of immune function is strongly dependent on the presence of the microbiota. One specific pathway connecting the gut microbiome and the brain operates via the vagus nerve. This route of communication is observed, for example, when infection of the gastrointestinal system with specific bacteria transmits abdominal immune information to the brain via that nerve [29]. Another connection between the gut microbiota and the brain occurs via neurotransmitters such as serotonin, the concentration of which is affected by the microbial metabolism of tryptophan, an essential amino acid and precursor to the neurotransmitter [30]. In this manner, the microbiome can affect gut–brain signaling by altering levels of tryptophan, kynurenine, serotonin and melatonin [31], all of which are necessary for maintaining homeostasis in gut–brain signaling. This metabolite-based route can affect MS-related processes, as demonstrated in the EAE animal model of MS; when EAE mice are fed a tryptophan-depleted diet, later dietary tryptophan supplementation results in improved clinical scores by tryptophan modulation of microglia [32].

**Figure 1.**
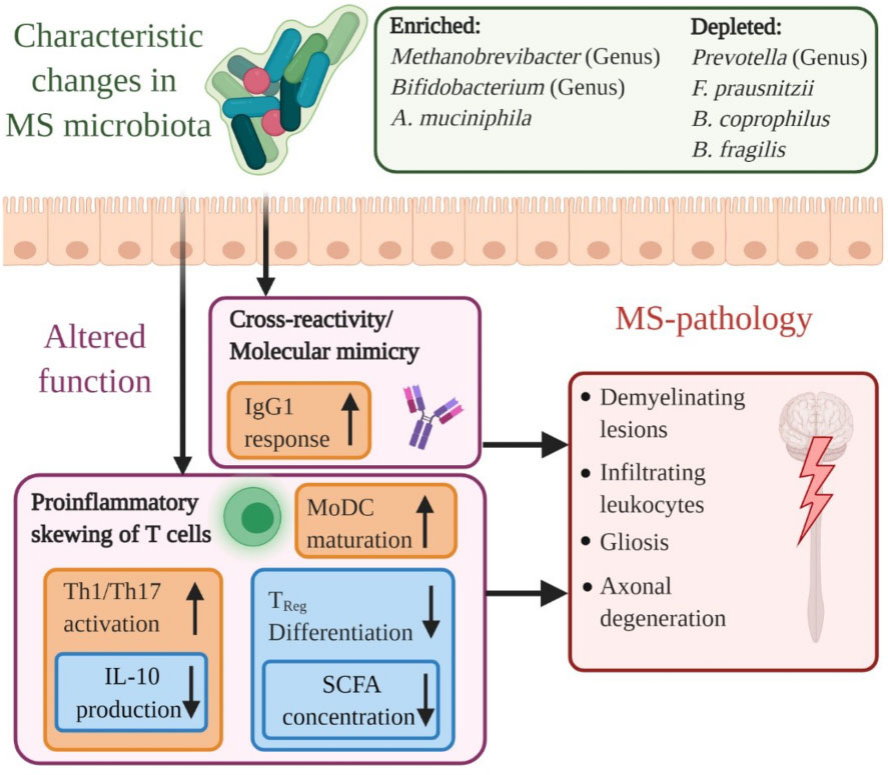
Hypothetical impact of the microbiome in the pathogenesis and progression of MS. Changes in the gut microbiome that are distinct to MS-patients influence immune function. Through these changes, a pro-inflammatory state manifests in the gut. Pro-inflammatory skewing of T cells and molecular mimicry affect the brain and spinal cord in MS

The microbiome could potentially also affect immune function and autoimmune diseases in a number of ways [33] (Fig. 1). One possible mechanism involves short chain fatty acids, particularly butyrate, which are synthesized in large part by the gut microbiota and can modulate regulatory T (Treg) cell function in the colon [34]. In addition, the gut microbiome can alter T helper cell activity, where pathobionts in the microbiome can create a more pro-inflammatory environment, rather than a tolerogenic environment [33]. Another way of shaping immune function through the microbiome is via a process called epitope spreading [33]. This happens when microbiota-related molecules closely resemble molecules expressed on host tissue. Unfortunately, due to this resemblance, immune reactivity against specific classes of microbes can lead to reactions to self, which in turn can lead to autoimmune disease. Such mechanisms of autoimmunity induction are e.g. associated with the presence of the *HLA-DRB1* gene, an immune-related gene that is the largest genetic risk factor for MS [18, 35]. Finally, the gut microbiome can be involved in autoimmune disease through a phenomenon where T cells can recognize both microbial and self-antigens through expression of two different T cell receptors, which provokes autoimmunity through activation by microbial antigens and subsequent reactivity to self-antigens [33]. To conclude, the gut microbiome can influence the immune system in various ways, possibly modulating autoimmune pathogenesis.

Any or all of the mechanisms described above, by which the microbiome and immune system interact, could potentially play a role in the etiology or pathophysiology of MS (Fig. 1). This view suggests that, potentially, the pathology of MS might be treated via modulation of microbial community composition, e.g. via specific dietary alterations. Some evidence that dietary interventions in MS might be effective has been obtained in the laboratory. For example, in an EAE mouse model, a western-style, salt-rich diet modulated the T helper cell 17 (Th17) axis through depletion of *Lactobacillusmurinus* and worsened the pathological course of disease. At the same time, administration of *L. murinus* ameliorated the clinical symptoms in EAE in the model [36]. Moreover, mice that were co-colonized with an *Erysipelotrichaceae* strain and a *Limosilactobacillusreuteri* strain had more severe EAE symptoms than germ-free mice or mice mono-colonized with *L. reuteri*, which is again indicative of microbial modulation of MS pathology [37]. Thus, to the extent that MS is mediated via microbiota, it is hoped that dietary interventions might be a safe, practical and effective means of developing new treatment options for MS.

## THE GUT MICROBIOME IN MS

Given that microbiota are necessary for the development of immune function, and that immune function is a prerequisite for autoimmune disease, it can be concluded that the microbiota could play a potentially important role in MS pathogenesis. However, whether that role is pivotal in the initiation and progression of disease remains unclear. With this in mind, differences between the microbiota of healthy controls and of patients with MS are of substantial interest. It is expected that, if the gut microbiota plays a pivotal role if the pathogenesis of MS, identifying features of the MS-associated microbiota will be observed. Fortunately, numerous studies have addressed this issue, as recently and expertly reviewed by Mirza and colleagues [22]. Commonly observed conditions in the microbiota of patients, through 16S rRNA sequencing of fecal samples, with MS are that species from the *Prevotella* genus are underrepresented compared to controls [22, 38–41], and *Akkermansia muciniphila* tends to be overrepresented [22, 38, 40, 42, 43].

However, numerous other differences between the microbiota of people with and without MS have been observed. For example, Cekanaviciute and colleagues found more *Acinetobacter calcoaceticus* and less *Parabacteroides distasonis* in patients with MS compared to controls [38]. As another example, Jangi and colleagues observed larger relative amounts of *Methanobrevibacter* and *Akkermansia*, less *Butyricimonas*, and, after immunomodulatory therapy, normalized amounts of *Prevotella* and *Sutterella* in patients with MS compared to healthy controls [40]. The idea that these differences might be important in the pathogenesis of MS (Fig. 1) is encouraged by the observation that all of those species have been implicated in the immunoregulatory response in humans [38].

However, as recently pointed out by Ghezzi and colleagues [44], differences found between the microbiota of patients with MS and of healthy controls are not consistent. Furthermore, differences between microbial metabolites identified from study-to-study are often inconsistent in part due to lack of large-scale randomized controlled trials [44]. Also, technical problems might contribute, to a certain extent, to difficulties in consistency between studies [45]. For instance, most microbial variance stems from a participant’s house and recruitment site [45]. Swidsinski and colleagues even go so far as to conclude that no changes in the microbiome can be identified that are specific for MS [46]. This lack of consistency suggests that no particular microbial composition is responsible for the pathogenesis of MS, an issue that could potentially complicate future efforts to modulate the microbiota for the purpose of treating MS.

Perhaps just as concerning as the inconsistency of observations regarding the microbiota of patients with MS, is the relatively small difference seen between the microbiota of patients with MS and of healthy controls in most studies. As pointed out by Swidsinski and colleagues, although some of the differences in the microbiota between patients with MS and controls reach the level of statistical significance, differences are moderate and can be influenced by a plethora of environmental factors that cannot be matched or sufficiently corrected for in a study design [46].

**Figure 2.**
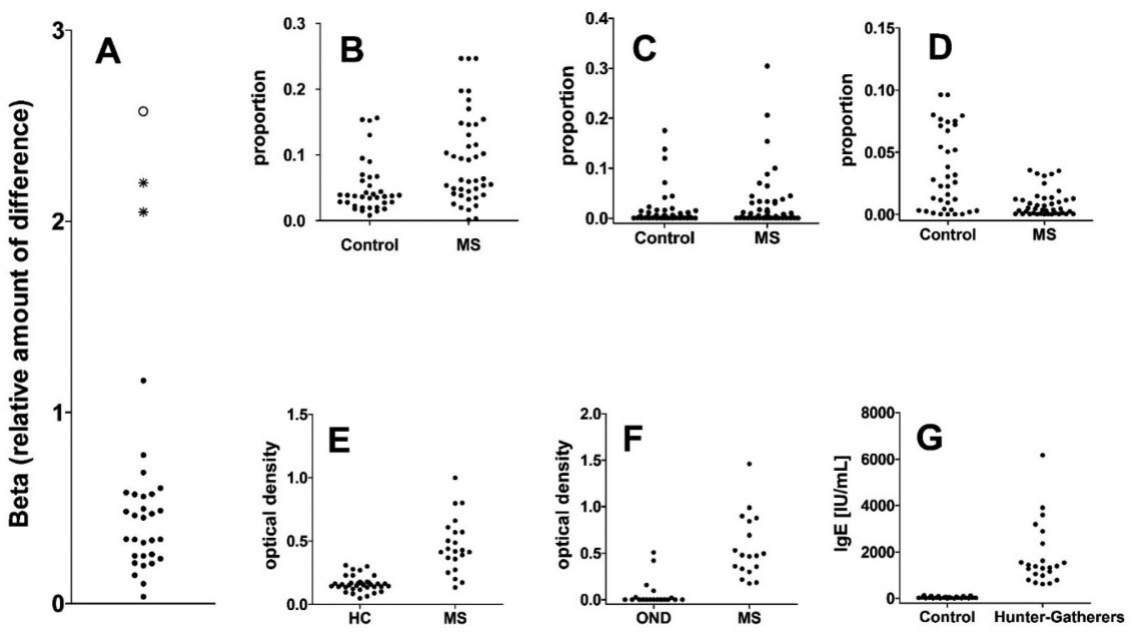
Differences in bacteria and in biomarkers between patients with multiple sclerosis and control groups. Data are taken from Table 1. Controls described in different studies included individuals without MS (Control), healthy controls (HC) or patients with other neurological disorders (OND). For comparison, IgE, a biomarker for exposure to helminths and protists, is shown in Western, non-allergic individuals versus hunter-gatherers. Panel (**A**) shows a summary of these differences using the value Beta = ratio of difference between the sample means divided by the average standard deviation. In that panel, each closed circle (all below Beta < 1.5) represents one type of bacteria, the two asterisks (between Beta = 2.0 and 2.5) represent the clinically useful biomarkers TCERG1 and HERV-W, and the open circle indicates IgE. Scatter plots are shown for three bacterial clades: (**B**) *Firmicutes-Blautia*, (**C**) *Akkermansia* (Beta = 0.257 from Table 1), and (**D**) *Bacteroidetes-Parabacteroides*. Scatter plots are also shown for (**E**) TCERG1, (**F**) HERV-W and (**G**) IgE

Our analysis of published data demonstrates that differences between the microbiota of individuals with MS and of healthy controls is observable only at the cohort level (Table 1, Fig. 2). That is to say, differences in average compositions can be identified when cohorts of individuals with and without MS are examined, but individual-to-individual variation is quantitatively greater than average differences between cohorts with and without MS. In marked contrast, clinically useful biomarkers for MS, such as TCERG1 and HERV-W, show differences between cohorts of patients with and without MS that exceed individual-to-individual variation (Table 1, Fig. 2). These observations further strain the hypothesis that microbiota plays a pivotal role in MS in humans, although it is possible that as yet unidentified features of the microbiota do play such a role.

**Table 1.** Differences in gut microbiota abundance and in various biomarkers between controls and patients with MS

Sample and sample size	Bacteria or biomarker	(Degree of overlap) Δ means/Average s.d.
*n* = 52 CTRL *n* = 52 MS [38]	*Akkermansia*	0.257
*n* = 36 CTRL *n* = 42 MS [39]	*Bacteroidetes-Parabacteroides*	1.167^a^
	Firmicutes-Blautia	0.777^a^
*n* = 43 CTRL *n* = 60 MS [40]	Euryarchaeota (454)	0.486
	Euryarchaeota (MiSeq)	0.571
	Verrucomicrobia (454)	0.250
	Verrucomicrobia (MiSeq)	0.481
	Methanobrevibacter (454)	0.461
	Methanobrevibacter (MiSeq)	0.560
	Akkermansia (454)	0.249
	Akkermansia (MiSeq)	0.449
	*Butyricimonas* (454)	0.604
	*Butyricimonas* (MiSeq)	0.470
	*Prevotella* (454)	0.235
	*Prevotella* (MiSeq)	0.331
	*Sutterella* (454)	0.103
	*Sutterella* (MiSeq)	0.035
	Sarcina (454)	0.198
	Sarcina (MiSeq)	0.212
*n* = 40 CTRL *n* = 20 MS [41]	Bifidobacterium	0.496
	Bacteroides	0.335
	Faecalibacterium	0.581
	Clostridium	0.210
	Ruminococcus	0.148
	Blautia	0.319
	Coriobacterium	0.337
	*Prevotella*	0.685
	*Streptococcus*	0.334
	*Anaerostipes*	0.573
*n* = 36 CTRL *n* = 22 MS [122]	HERV-W	2.202
*n* = 19 OND *n* = 18 MS [123]	ELISA Sera vs TCERG1	2.049
*n* = 25 CTRL [124] *n* = 23 Hunt-Gather [125]	IgE	2.576

a The data are taken from Fig. 3D and E of the reference cited. In that manuscript, the study was restricted to patients with relapsing-remitting MS (*n* = 31), except for Fig. 3D and E, in which case additional patients with MS (e.g. patients with primary-progressive MS and secondary-progressive MS) were included (Personal communication from Dr Ashutosh K. Mangalam, used with permission).

CTRL, control; ELISA enzyme linked immunosorbent assay; Gather, gatherer; HERV, human endogenous retrovirus; Hunt, hunter; IgE, immunoglobulin E; MS, multiple sclerosis; s.d., standard deviation; TCERG1, transcription elongation and splicing factor.

Several investigators have noted that effective treatment of MS using immunomodulatory drugs (IMDs) eliminates some of the major differences between the microbiota of individuals with MS and individuals without MS. For example, lower levels of *Prevotella* and *Sutterella* were found in patients with untreated MS, but not in patients treated with IMDs [40], indicating that treatment of immune dysfunction effectively reversed the association between MS and microbial community abnormalities in the gut. Furthermore, early results from an ongoing study suggest that levels of *A. muciniphila* and *Faecalibacteriumprausnitzii* are reduced to levels matching healthy controls following treatment with IMDs [43]. Notably, the observation that treatment of immune dysfunction effectively eliminates associations between MS and the microbiota, strongly suggests that alterations in the microbiota associated with MS are a result of the disease, not a cause (Fig. 3).

**Figure 3.**
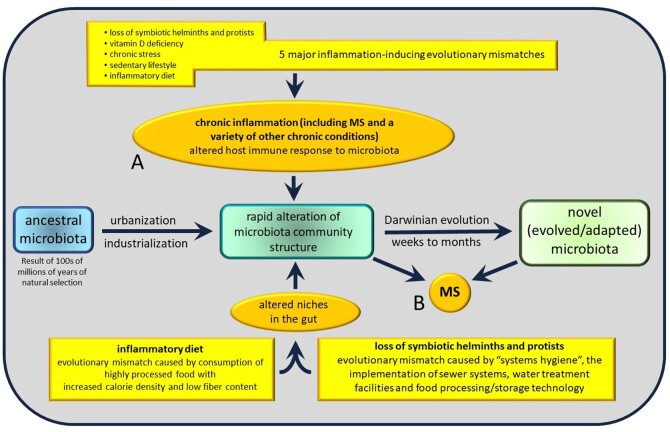
Relationships between diet, evolutionary mismatch, the gut microbiota and MS. In this diagram, two possible models for the induction of MS are shown. In (**A**), a variety of evolutionary mismatches lead to changes in the microbiota as well as a variety of chronic inflammatory diseases, including MS. In this model, MS is not directly induced by changes in the microbiota. In (**B**), MS occurs as a result of changes in the microbiota that are caused by evolutionary mismatches. Although it is possible that either one or even both models could apply in any given case, the preponderance of evidence supports model (A) as the predominant mechanism by which MS is associated with changes in the microbiota

## DIET, THE MICROBIOTA AND MS

Attempts to alter the progression of MS using dietary modifications predate our current appreciation of the associations between MS and the gut microbiota [16, 17]. However, the associations between MS and alterations in the microbiota (described above) and the fact that nutritional profiles do shape the gut-microbiome [47–49], have renewed and increased interest in the impact of diet on MS. Several different types of dietary interventions have been investigated (Box 5), including: (i) the Mediterranean diet, consisting of fish, monounsaturated fats from olive oil, vegetables, whole grains and legumes/nuts [50], (ii) high fiber diets, (iii) the paleolithic diet, based on the estimated food-intake of ancient hunter-gatherers, consisting mainly of vegetables, fruits, nuts, seeds, eggs, fish and lean meat, with little grains, dairy, salt and refined sugar [51], and (iv) low fat diets. Importantly, all of these diets have some things in common, including the consumption of food with lower calorie density and more fiber than the typical Western diet.

Numerious lines of evidence indicate that diet has an impact on the progression of MS (Box 6). Although studies on the effects of diet on MS tend to be small and some are lacking key control arms, most diets show at least some promise. For example, in a pilot study examining the effects of a Mediterranean diet on MS, adherence to the diet ameliorated fatigue and MS symptoms and disability [52]. In a pilot study evaluating the effects of a high-vegetable/low-protein diet on MS, the progression of disability and relapse rate were reduced in people adhering to this particular diet instead of a western diet [53]. Adherence to the Wahls diet, a modified paleolithic diet, lessened fatigue in MS patients [54], and, in a separate trial, showed improvements in perceived fatigue and physical and mental health [55]. Adherence to the McDougall diet, a low-fat diet, led to a decrease in fatigue, and a better BMI and blood-lipid profile in patients with MS [56].

Swank and Goodwin have summarized previous studies regarding the effects of another low-fat diet, the Swank diet, on MS [57]. Within a group of 144 patients with MS, 70 adhered very well to this diet. Mortality over a 34-year span was dramatically decreased in the group that adhered well to the Swank diet (20%) in comparison to patients who adhered badly to the Swank diet (61%). The age of patients at the beginning of the study was between 30 and 42 years, and deaths were recorded only if attributed to MS. However, as groups were defined based on adherence to the diet, there was no randomization, and confounding factors, such as an overall healthier lifestyle in people with high adherence rates, could not be excluded [57]. One small study showed some potential clinical benefit of a ketogenic diet, as the diet improved overall quality of life and a reduction in lymphocyte count and disability [120].

The effects of specific dietary supplements on MS have also been investigated. As with studies on diet and MS, these studies tend to be small and are often lacking key control arms. Specific supplements tested include long chain polyunsaturated fatty acids (LCPUFA), biotin, isoflavones and vitamin D. As with dietary changes, dietary supplements tend to show some promise in at least some studies. For example, although supplementation with ω-3 LCPUFA did not appear to have any beneficial effect for patients with MS [59], supplementation with ω-3 and ω-6 LCPUFAs together, reduced annual relapse rate and MS disease progression [60]. Furthermore, the intake of a high dose of biotin was related to an improvement of MS symptoms related to the spinal cord [61]. Similarly, in a double-blind randomized controlled study, a high-dose biotin supplement reduced the proportion of patients that progressed in their disability scores [62]. Moreover, an isoflavone diet attenuated EAE through the presence of isoflavone-metabolizing bacteria [63]. Furthermore, in patients with low vitamin D levels, vitamin D supplementation was associated with a lower annual relapse rate, a reduction in brain lesions and a slower progression of disability [64]. However, high-dose supplementation of vitamin D did not improve the proportion of patients without disease activity or with new T1-weighted lesions. Still, a reduction in total volume of T2-weighted lesions was observed [65]. This suggests that supplementary vitamin D is beneficial for MS patients who are experiencing vitamin D insufficiencies.

The effects of the various diets and dietary supplements on the gut microbiota community composition are summarized in Table 2. In general, the effects of diet and dietary supplements on the microbiota cover a wide range of organisms, are dependent on the particular diet or supplement and are not obviously related to the MS-associated abnormalities in the microbiota. For example, beneficial effects of conjugated linoleic acid in an EAE model were not dependent on the microbiome but rather acted on myeloid cells in the gut directly [66]. In at least one case, the relationship between diet, MS and the microbiota is not what might be expected; Enrichment of species from the *Akkermansia* genus, particularly *A. muciniphila*, is sometimes observed as a characteristic of the MS-associated microbiota [22], but nutritional interventions such as a paleolithic or ketogenic diet are also associated with an increase of the *Akkermansia* genus, particularly *A. muciniphila* [51]. Moreover, the gut microbiota is typically studied through fecal samples, whereas nutrient absorption is more pronounced in the small intestine, as is the case for dietary biotin [67]. This discrepancy could explain the relatively small and varying effect of nutritional interventions on studied gut microbiota.

**Table 2.** Dietary interventions in MS

Nutritional intervention	Selected microbiome changes	Functional impact	Clinical trials
*Mediterranean diet*	*F. prausnitzii ↑* [126]	SCFA ↑ [127]	Improved fatigue and MS symptom impact [52]
*Dietary fiber supplement*	*Lachnospiraceae ↑* *Bifidobacterium↑* [53, 128]	Treg ↑ TGF-β ↑ IL-10 ↑ [53]	Slowed EDSS progression [53]
*SCFA supplements*	*Firmicutes: Bacteroides ↓* [106]	Treg ↑ IL-10 ↑ Th1/Th17 ↓ [105, 129]	Reduced brain atrophy, disease progression and relapse rate [105]
*LCPUFA* *supplements*	*Faecalibacterium ↕* *Bifidobacterium ↑* *Roseburia ↑* [130]	Th1/Th17 ↓ [131]	PLP10 supplement: Reduced relapse rate and disease progression [60]
*Paleolithic diet*	*Akkermansia* ↑ [51]	Unknown	Improved fatigue [54, 55]
*Low fat diet*	*Prevotella ↓* *Blautia ↓* [132]	Unknown	Improved fatigue [56]
*Ketogenic diet*	*muciniphila* ↑ *Bifidobacterium* ↓ *Faecalibacterium* spp. ↓ [133–135] *Normalized MS gut microbiome* [46]	Unknown	No clinical benefit [136] Improved fatigue [137] Improved inflammatory status [58, 138] Decreased EDSS [58]
*Biotin supplement*	*L. murinus* ↑ [139]	Protection against hypoxia [61]	Improvement in spinal cord [61] Slowed EDSS progression [62]
*Vitamin D supplement*	*Prevotella ↕* *Bifidobacterium* ↓ [140]	Treg ↑ Th1/Th17 ↓ [141]	Lower disease progression in vitamin D insufficient patients [64] No benefit of high-dose over low-dose [142]
Summary of changes due to nutritional interventions as compared to control groups. Articles were collected through Google Scholar, PubMed, Web of Science, clinicaltrials.gov and EudraCT. Search terms such as the relevant nutritional interventions in combination with ‘Microbiome’, ‘Microbiota’, ‘Multiple Sclerosis’ and ‘Clinical Trial’ were used. EDSS = Expanded Disability Status Scale.			

The observations that all of these diets and supplements (i) are generally improvements upon the Western diet that are expected to increase overall gut health, (ii) show some promise of alleviating some aspects of MS, and (iii) have varying effects on the microbiota (Table 2) has important implications: it seems likely that healthy improvements in diet without regard for particular microbial community compositions might be beneficial for MS. This idea adds further weight to the view that the alterations in the microbiota associated with MS are a result of MS or factors that cause MS, not a cause of MS (Fig. 3).

## EVOLUTION AND THE BIOTA AND INTRODUCTION OF MISMATCHES

**Figure 4.**
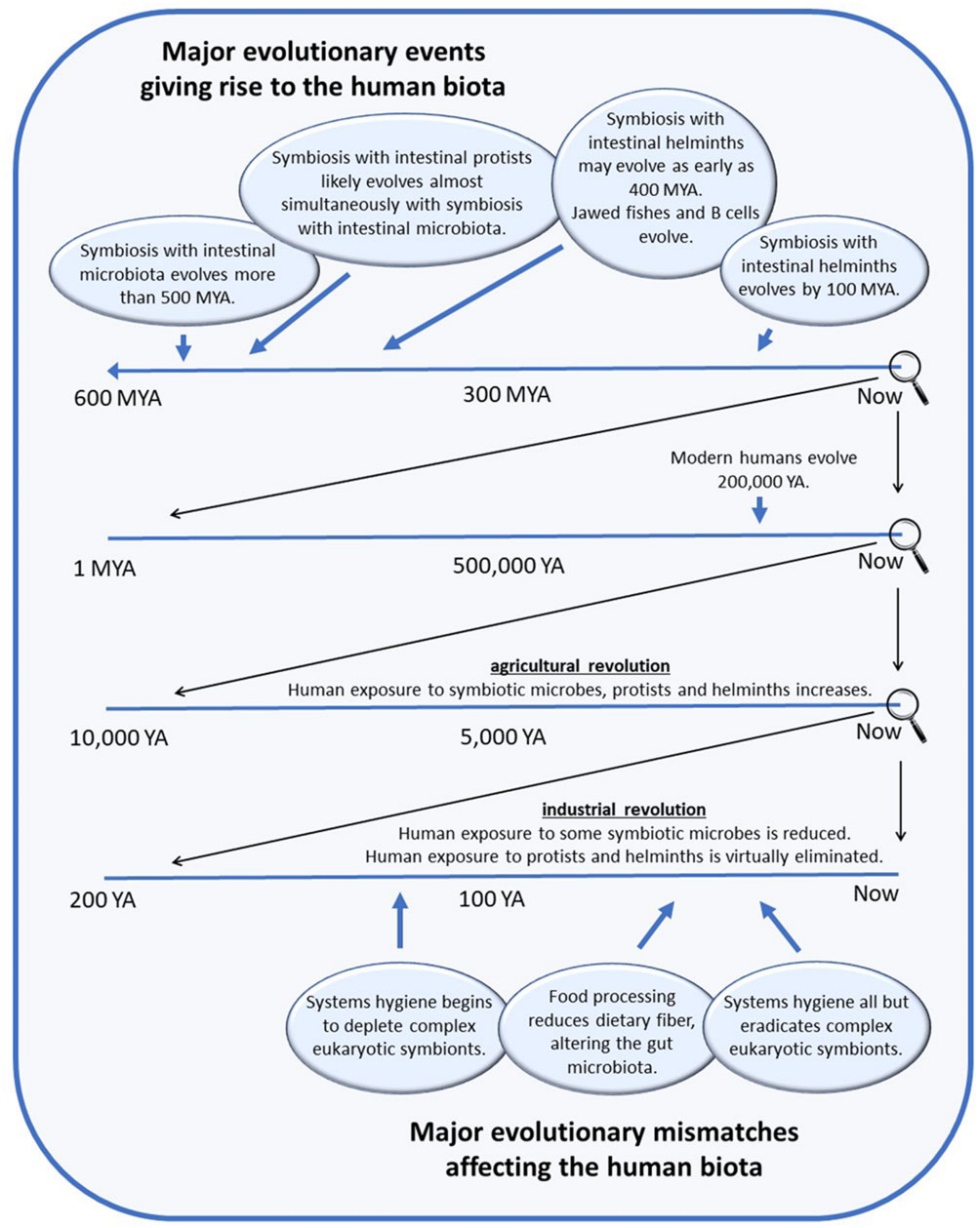
Evolution of symbiotic relationships of the gut and introduction of mismatches affecting those relationships. In this diagram, a cascading series of timelines are shown, with lowest magnification, starting at 600 MYA (million years ago) on top. The timeline with the highest magnification, starting at 200 YA (years ago) is shown on the bottom. The approach used to estimate time of appearance of various symbiotic relationships during evolutionary history (top timeline) in the gut is described in the text. The evolutionary mismatches shown in the bottom timeline apply to communities with widespread systems hygiene [11] and to individuals with highly processed diets [49]

When considering the evolutionary mismatches that may at least in part underlie autoimmune disease, including MS, it is important to consider the evolution of host species with the entire gut biota, which includes both the microbiota and complex eukaryotic symbionts such as helminths and protists. An estimated timeline of evolution of gut symbiosis is shown in Fig. 4. The symbiosis of animals with their gut microbiota may be almost as old as the origins of the gut itself. The evolution of diploblasty, the presence of two tissue layers, facilitated the formation of the first true guts more than 500 million years ago [68]. The hydra, a simple diploblastic animal composed by only 50 000–100 000 cells, can be described as a digestive tract with an anchoring foot and a tentacle-lined mouth [69]. Despite their simplicity, hydras host a gut microbiome and maintain that microbiome using molecular mechanisms analogous to some of those found in mammals [70]. It is widely accepted that these such diploblastic animals gave rise to triploblastic animals such as humans and fruit flies. Thus, it is possible that the symbiosis of humans with their microbiota may have an evolutionary history dating back more than 500 million years, predating the evolution of triploblasty and chordates.

The origins of the symbiosis of animals with intestinal protists may be difficult to assess. However, the free-living protist *Tetrahymena* is known to colonize the aforementioned hydra [71], a simple diploblastic animal. Thus, protists can colonize the guts of the simplest organisms known to contain a gut microbiome, suggesting that the evolution of symbiosis between animals and intestinal protists could have origins as ancient as the intestinal microbiota itself (Fig. 4). The symbiosis of animals with intestinal helminths is also ancient, although not as ancient as the symbiosis between animals with their microbiome. Both classes of intestinal helminths, cestodes and nematodes, are triploblastic, having evolved after the first diploblastic organisms that probably contained symbiotic microbiota. Several investigators have made efforts to determine how long helminths have been associated with the guts of animals. The consensus is that vertebrates have been living with symbiotic intestinal helminths for at least 100–200 million years, but such symbiosis could go back 400 million years [72] when T cells and B cells first diverged with the evolution of jawed fish [73].

Based on the appearance of symbiotic relationships within the gut as well as mismatches affecting those relationships (Fig. 4), two important conclusions can be drawn. First, modern evolutionary mismatches that alter gut symbiosis, including changes in diet and widespread use of systems hygiene, have occurred on a timescale that is ∼ 6 million-fold faster than the evolution of the symbiotic relationships themselves. Second, evolutionary mismatch has most profoundly altered complex eukaryotic symbionts whose evolutionary relationship with the gut is probably almost as old as the gut itself (Fig. 4). Although highly processed diets have altered the microbiota to a large extent, human hosts have adapted to a wide range of microbiota that correspond to varying diets, including a very low-fiber consumption among hunter-gatherer individuals with a predominantly meat-based diet [74]. Furthermore, even a diet completely devoid of all dietary fiber due to food processing, an extreme situation that only occurs in controlled laboratory settings, still facilitates the presence of a complex microbiota [75]. Thus, a consideration of the evolution of symbiotic relationships along with corresponding evolutionary mismatches supports the view that modern, diet-based alterations in the microbiota are not the most prominent change to the biota imposed by a Western lifestyle. Rather, it is the almost complete loss of complex eukaryotic symbionts due to systems hygiene that constitutes the most substantial change imposed on the biota by a Western lifestyle. Given the ancient origins of our symbiosis with complex eukaryotic symbionts (Fig. 4), their recent loss constitutes a potentially dangerous evolutionary mismatch of considerable concern.

Brian Greenwood noted more than 50 years ago that the absence of complex eukaryotic symbionts, including helminths and protists, was the single most important factor affecting immune function and the increasing prevalence of autoimmune disease in the Western society [7–10]. For comparative purposes, a marker of exposure to complex eukaryotic symbionts is shown in Fig. 2G. As can be seen, the absence of complex eukaryotic symbionts causes a very profound shift in immune markers (Fig. 2, Table 1). Work by Correale from Argentina [5, 6, 76] as well as our own studies [77, 78] show that re-introduction of complex eukaryotic symbionts halts the progression of (relapsing–remitting) MS, for example through direct modulation of the host immune system [76]. This provides conclusive evidence supporting the idea that loss of eukaryotic symbionts is *the* pivotal evolutionary mismatch that underlies the pathogenesis and progression of MS.

Further evidence for the importance of complex eukaryotic symbionts in the etiology of MS comes from emerging data supporting the view that infection with the Epstein–Barr virus (EBV) can trigger MS [79]. Although EBV apparently has ‘no effect on microbiome composition whatsoever’ [80], adverse reactions to a wide range of viral infections are apparently a consequence of the loss of complex eukaryotic symbionts [11, 72, 81]. Such mismatch-facilitated adverse reactions include the triggering of autoimmune disease [11, 72], pointing again to the loss of complex eukaryotic symbionts as the critical mismatch involved in the onset and pathogenesis of MS. In this scenario, viral infections potentially act as a trigger for disease, and the loss of complex eukaryotic symbionts acts as an evolutionary mismatch and necessary ‘cofactor’ for the induction of disease [72]. Nevertheless, studies thus far have focused on the microbiota of fecal samples in patients with MS rather than the microbiota of the small bowel in those patients. With this in mind, we cannot rule out the potential importance of the small bowel microbiota in the intersection of diet and MS.

## FUTURE PERSPECTIVES

This narrative review addresses several key issues that provide insight into some of the possible evolutionary mismatches underlying the connection between the microbiota and MS. Two lines of evidence support the view that the microbiota may be important in the pathogenesis and progression of MS. First, studies in animal models support the idea that the microbiota is involved in the pathogenesis of the disease. Second, in cohorts of human subjects with MS, alterations in the microbiota have been observed, and these alterations may, based on our current understanding of the effects of the microbiota on immune function, be involved in the pathogenesis and progression of MS.

This narrative also describes a number of key issues that strongly discourage the view that the microbiota alone play a pivotal role in the initiation and progression of MS. First, MS-associated features of the gut microbial community composition are typically not consistent from study-to-study and may depend on the cohort evaluated. More concerning is the observation that individual-to-individual variations appear to overshadow trends in MS-associated variations in the microbiota. In addition, treatment of MS via immunomodulation tends to reverse many of the associations of MS with an altered microbiota, suggesting that alterations in the microbiota are an effect rather than a cause of the disease.

Furthermore, preliminary studies suggest that health-oriented adjustments to nutritional status, be it by altered diet or by the addition of supplements, can possibly help alleviate the progression of MS, but do not alter the microbiome in such a way as to reverse MS-associated features of the microbiota. Diet is the primary mediator of microbial community composition within the human gut [82]. Thus, the view that diet does not reverse MS-associated alterations to the microbiota suggests that the MS-associated alterations to microbiota are not the driving force behind the initiation and progression of MS. Finally, considering the evolution of symbiotic relationships in the intestine as well as the introduction of mismatches affecting those relationships, it is expected that autoimmune conditions such as MS are heavily influenced by evolutionary mismatches affecting complex eukaryotic symbionts rather than evolutionary mismatches affecting the gut microbiota.

We suggest that, given the critical role played by the microbiota in the development of the immune system, microbiota may play a critical role in all immune processes, including autoimmune diseases. However, at the same time, we argue that the gut microbiota of the large bowel, which includes prokaryotic organisms as well as fungal species, does *not* appear to play the pivotal role in the pathogenesis or progression of MS, and that evolutionary mismatches associated with the general health of the immune system are probably more important in that regard (Fig. 3). Analogous situations are evident throughout biology and medicine. For example, as much as growth factors play a critical role in the development of cardiovascular systems, those factors are not the pivotal players that determine whether an individual develops arteriosclerosis. Rather, it is evolutionary mismatches such as culture-associated stress, diet and sedentary lifestyle that are the key players in the pathogenesis and progression of arteriosclerosis.

Based on available evidence, we conclude that the benefits of improved diets and of dietary supplements to MS patients, probably act through improved health and immune functions rather than via pathways associated with the microbiota–immune system axis. Regardless of this conclusion, healthy diets are still strongly encouraged for patients with MS. At the same time, this conclusion suggests that microbiota-targeted therapies for MS may be less effective than other approaches currently in use. Difficulties with microbiota-targeted therapies are further hindered by the rapid evolution of microbes under laboratory conditions [49], a factor that will likely impede all attempts to develop and maintain a stable microbiota-based product in the laboratory. With these considerations in mind, we advocate for continued work aimed at encouraging a healthy diet in the MS and other populations, and for increased efforts to address contributions from other evolutionary mismatches that lead to autoimmune disease as well as other chronic inflammatory conditions.

**Running Glossary:** The Running Glossary is provided in: Box 3. Key definitions.
